# Loss of the SPHF Homologue Slr1768 Leads to a Catastrophic Failure in the Maintenance of Thylakoid Membranes in *Synechocystis* sp. PCC 6803

**DOI:** 10.1371/journal.pone.0019625

**Published:** 2011-05-23

**Authors:** Samantha J. Bryan, Nigel J. Burroughs, Carol Evered, Joanna Sacharz, Anja Nenninger, Conrad W. Mullineaux, Edward M. Spence

**Affiliations:** 1 School of Biological and Chemical Sciences, Queen Mary University of London, London, United Kingdom; 2 Systems Biology Centre, University of Warwick, Coventry, United Kingdom; 3 Warwick HRI, Wellesbourne, Warwick, Warwickshire, United Kingdom; 4 Department of Biological Sciences, University of Warwick, Coventry, United Kingdom; Netherlands Institute of Ecology, The Netherlands

## Abstract

**Background:**

In cyanobacteria the photosystems are localised to, and maintained in, specialist membranes called the thylakoids. The mechanism driving the biogenesis of the thylakoid membranes is still an open question, with only two potential biogenesis factors, Vipp1 and Alb3 currently identified.

**Methodology/Principal Findings:**

We generated a *slr1768* knockout using the pGEM T-easy vector and REDIRECT. By comparing growth and pigment content (chlorophyll *a* fluoresence) of the Δ*slr1768* mutant with the wild-type, we found that Δ*slr1768* has a conditional phenotype; specifically under high light conditions (130 µmol m^−2^ s^−1^) thylakoid biogenesis is disrupted leading to cell death on a scale of days. The thylakoids show considerable disruption, with loss of both structure and density, while chlorophyll *a* density decreases with the loss of thylakoids, although photosynthetic efficiency is unaffected. Under low light (30 µmol m^−2^ s^−1^) the phenotype is significantly reduced, with a growth rate similar to the wild-type and only a low frequency of cells with evident thylakoid disruption.

**Conclusions/Significance:**

This is the first example of a gene that affects the maintenance of the thylakoid membranes specifically under high light, and which displays a phenotype dependent on light intensity. Our results demonstrate that Slr1768 has a leading role in acclimatisation, linking light damage with maintenance of the thylakoids.

## Introduction

Nearly all cyanobacteria possess thylakoid membranes, the single known exception being the atypical cyanobacterium *Gloeobacter* [1], which houses all its photosynthetic complexes in the plasma membrane. Cyanobacterial thylakoids have a complex architecture [2], and in contrast to chloroplasts are not stacked, instead being organised as either concentric shells around the cell or in parallel sheets. They are densely packed with photosynthetic complexes, and have a protein composition quite distinct from that of the plasma membrane [3]. The mechanisms governing thylakoid biogenesis are poorly understood in both cyanobacteria and chloroplasts. Several models for thylakoid biogenesis have been put forward. The two favoured scenarios suggesting that components are either, synthesised and assembled in specialised thylakoid regions or that initial production of polypeptides and assembly of protein/pigment complexes occurs in the plasma membrane, with these precomplexes being transferred to the thylakoids via a yet unidentified mechanism [4]. Thylakoid membranes do appear to converge on the plasma membrane at specific sites; these sites are thought to mark thylakoid centres where thylakoid biogenesis is initiated [5], [6]. Membrane fractionation studies have suggested that precomplexes of both photosytems are indeed assembled within the plasma membrane [7]. Chloroplasts develop from proplastids and it is assumed that thylakoid membranes formed during the maturation process are derived from the inner envelope [8]. No internal membrane systems are described for any other eukaryotic organelle; it is therefore tempting to speculate that the biogenesis of the thylakoid membrane is directly coupled to the development of oxygenic photosynthesis [9]. Mutant studies in *Arabidopsis thaliana* and *Synechocystis* sp. PCC 6803 have identified two key genes, which affect thylakoid development both directly and indirectly, namely Vipp1 (Vesicle-inducing protein in plastids 1), and Alb3. Both Alb3 (*slr1471*) and Vipp1 (*sll0617*) were first identified in chloroplasts via insertional mutagenesis [10], [11] and like Slr1768 are both located in the plasma membrane, although Vipp1 has also been detected in the thylakoid membrane [12]. The *Arabidopsis* Δ*vipp1* mutant, in which the *vipp1* gene disruption resulted in the abolition of vesicular transport in chloroplasts, was shown to be essential for thylakoid biogenesis. Recent research utilising an *Arabidopsis* Δ*vipp1* mutant, which had been engineered to produce reduced amounts of a Vipp1-ProteinA fusion protein, allowed a demonstration that although there was a loss in the photosynthetic performance, this loss was due to an overall decrease in the thylakoid membrane and not due to the loss of photosynthetic protein insertion or assembly directly [13]. This suggests that in *Arabidopsis*, Vipp1 plays a role in basic thylakoid membrane formation, and not in the functional assembly of thylakoid protein complexes [13]. In cyanobacteria deletion of *vipp1* also leads to a loss in thylakoid content [9], however the Δ*vipp1* mutant does not fully segregate, showing that Vipp1 is essential. A recent study has implicated Vipp1 as having a role in PS1 assembly and stability in *Synechocystis* sp. PCC 6803 [14]. Fuhrmann *et al*. demonstrated that thylakoid numbers decreased in a *vipp1* mutant but, interestingly, they were unable to isolate a strain with no thylakoids, suggesting that a *vipp1* deletion results in a reduction in the thylakoid membrane, as opposed to abolishing thylakoid biogenesis altogether. Further analysis of the photosynthetic complexes indicated a significant reduction and destabilisation of the PS1 trimers in the *vipp1* mutant [14].

The chloroplast Alb3 homologue is required for the insertion of the light harvesting chlorophyll binding proteins (Lhcb) into the chloroplast thylakoid membrane [15]. A second albino homologue Alb4 has been shown to be essential for chloroplast biogenesis in both *Arabidopsis* [16] and *Chlamydomonas* [17]. In *Synechocystis*, the Alb3 homologue (*slr1471*) is essential for thylakoid biogenesis [18], and it has also been shown to be necessary for the insertion of the D1 protein, an essential photosystem II core protein [19]. Therefore thylakoid biogenesis mutants have so far fallen into two categories, either those which appear to directly affect thylakoid biogenesis (Vipp1), or those which result in impaired insertion of essential photosynthetic proteins into the thylakoid membrane (Alb3 and Alb4).

In this paper we report a third thylakoid biogenesis regulator, Slr1768. During a mutagenesis screen of prohibitin homologues in *Synechocystis* sp. PCC 6803 we observed that disruption of the gene *slr1768* had a particularly interesting and unexpected phenotype relating to thylakoid biogenesis. BlastP analysis demonstrated that Slr1768 is an HflC homologue, and a member of the prohibitin family. Prohibitins comprise an evolutionarily conserved and ubiquitously expressed family of membrane proteins which have various roles in different cellular compartments. These include transcriptional regulation, cellular signalling, apoptosis and mitochondrial biogenesis [20]. In *E. coli* the HflK and HflC proteins form a large complex [21], which affects the lysis/lysogeny decision of bacteriophage lambda, with mutants demonstrating a high frequency of lysogeny (HFL) [22], [23], [24]. Recent evidence has also shown that HflK and HflC regulate the membrane protease FtsH in *E. coli* [25], which is responsible for the degradation of misfolded or damaged membrane proteins including the λCI repressor [26]. Genes encoding Slr1768-like proteins have been found in both prokaryotes and eukaryotes including plants and humans. In this paper, we demonstrate that disruption of the *slr1768* gene in *Synechocystis* leads to a malfunction in thylakoid membrane formation that is only seen occasionally under low light. However, the phenotype is greatly exacerbated under high light conditions. PSII electron transport efficiency seems to remain undiminished in those cells which retain their pigment. This indicates a critical role for Slr1768 in the maintenance of thylakoid membranes, under high light.

## Results

### Deletion of *slr1768* in *Synechocystis* sp. PCC 6803

To investigate the role of Slr1768 in *Synechocystis* sp. PCC 6803, we generated a full in-frame deletion by the insertion of a spectinomycin resistance cassette into the wild type gene using the REDIRECT gene disruption protocol [27]. The gene, including 1kb of flanking genomic DNA either side, was amplified using the primers slr1768F and slr1768R and cloned into the pGEM T-easy vector, to generate pG1768. Successful colonies were screened via PCR to confirm the presence of the 3 kb insert and sequenced. The spectinomycin resistance cassette was then introduced into pG1768 as detailed in Gust *et al*., 2003. An analysis of the adjacent ORFs, Sll1638, encoding a hypothetical protein, and Sll1636, which encodes a ferripyochelin binding protein, using the web-tool NNPP, suggests that each have their own promoter and, are likely to be transcribed independently. Hence, insertional inactivation of Slr1768 should not lead to polar effects on downstream genes. Wild-type *Synechocystis* cells were transformed with the construct pG1768 and grown on plates in the presence of spectinomycin as detailed in Materials and Methods. A *slr1768* mutant was obtained by homologous recombination and full disruption of the gene was confirmed by PCR and by Southern blotting (data not shown), demonstrating insertion of the spectinomycin-resistance cassette into the *slr1768* gene. Full segregation was confirmed via PCR using both wild-type and Δ*slr1768* genomic DNA (data not shown).

### Disruption of *slr1768* in *Synechocystis* sp. PCC 6803 leads to a light-dependent loss in pigment content and growth rate

**Figure 1:**
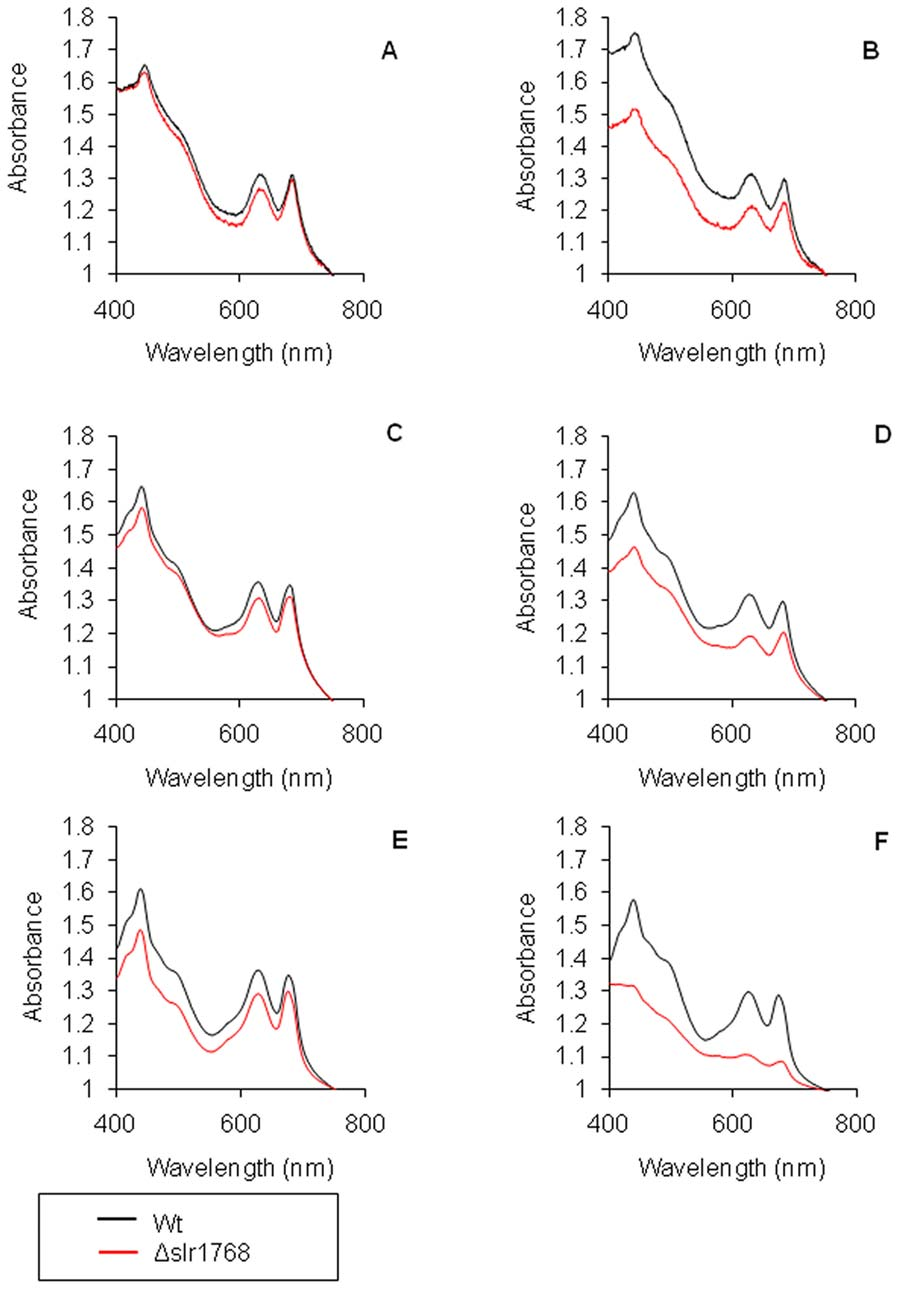
Photoautotrophically grown *slr1768* cells exhibit a loss of pigment. Black  = WT and red = Δ*slr1768*. A, C and E whole cell absorption spectrum of wild-type and Δ*slr1768* cells grown under low light (30 µmol m^−2^⋅s^−1^) at 15 hours (A) 48 hours (C) and 90 hours (E). Similarly, B, D and F grown under high light conditions (130 µmol m^−2^ s^−1^) at 15 hours (B), 48 hours (D) and 90 hours (F). All samples were normalised to OD_750_.

**Figure 2:**
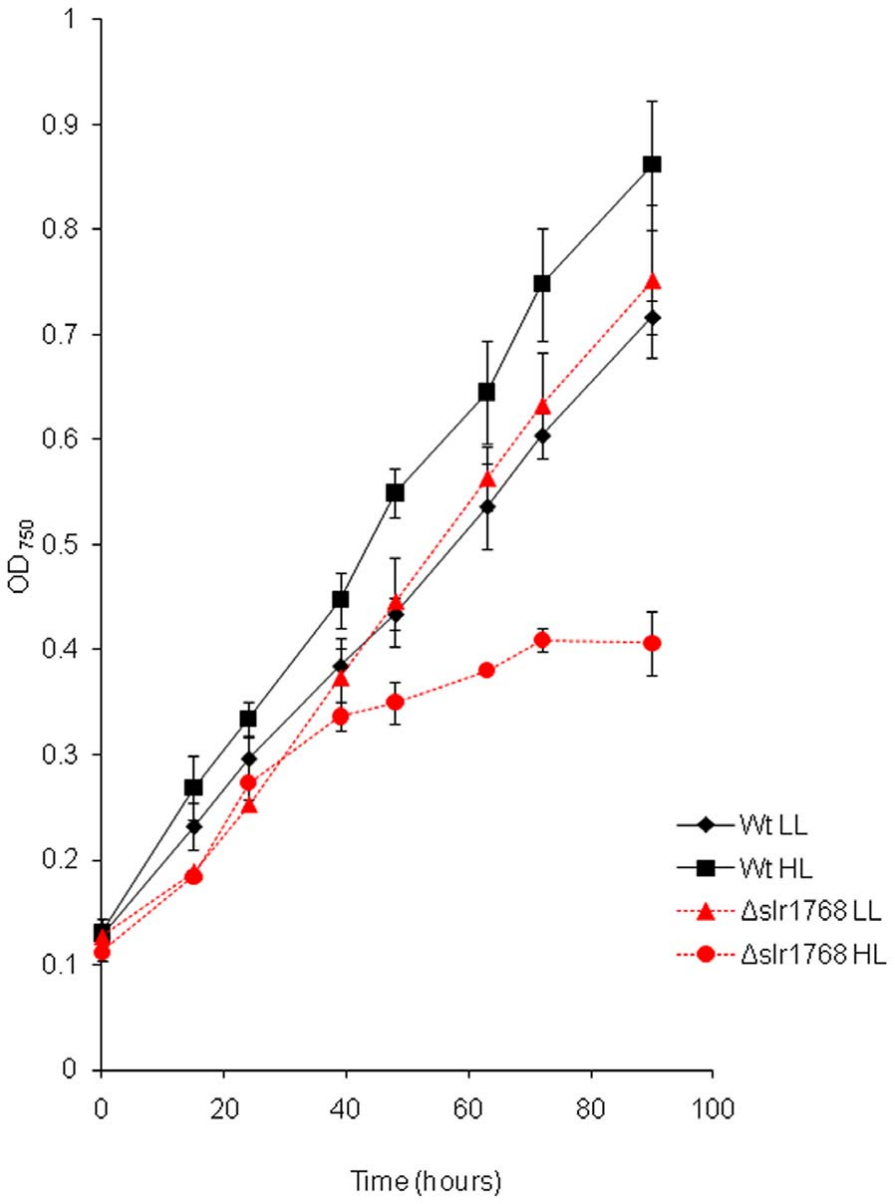
Photoautotrophically grown Δ*slr1768* cells under low and high light. Δ*slr1768* cells grown under high light (130 µmol m^−2^ s^−1^) show a cessation in active growth. Graph shows growth of wild-type and Δ*slr1768* cells over 90 hours under both high light (130 µmol m^−2^ s^−1^) and low light (30 µmol m^−2^ s^−1^).

**Figure 3:**
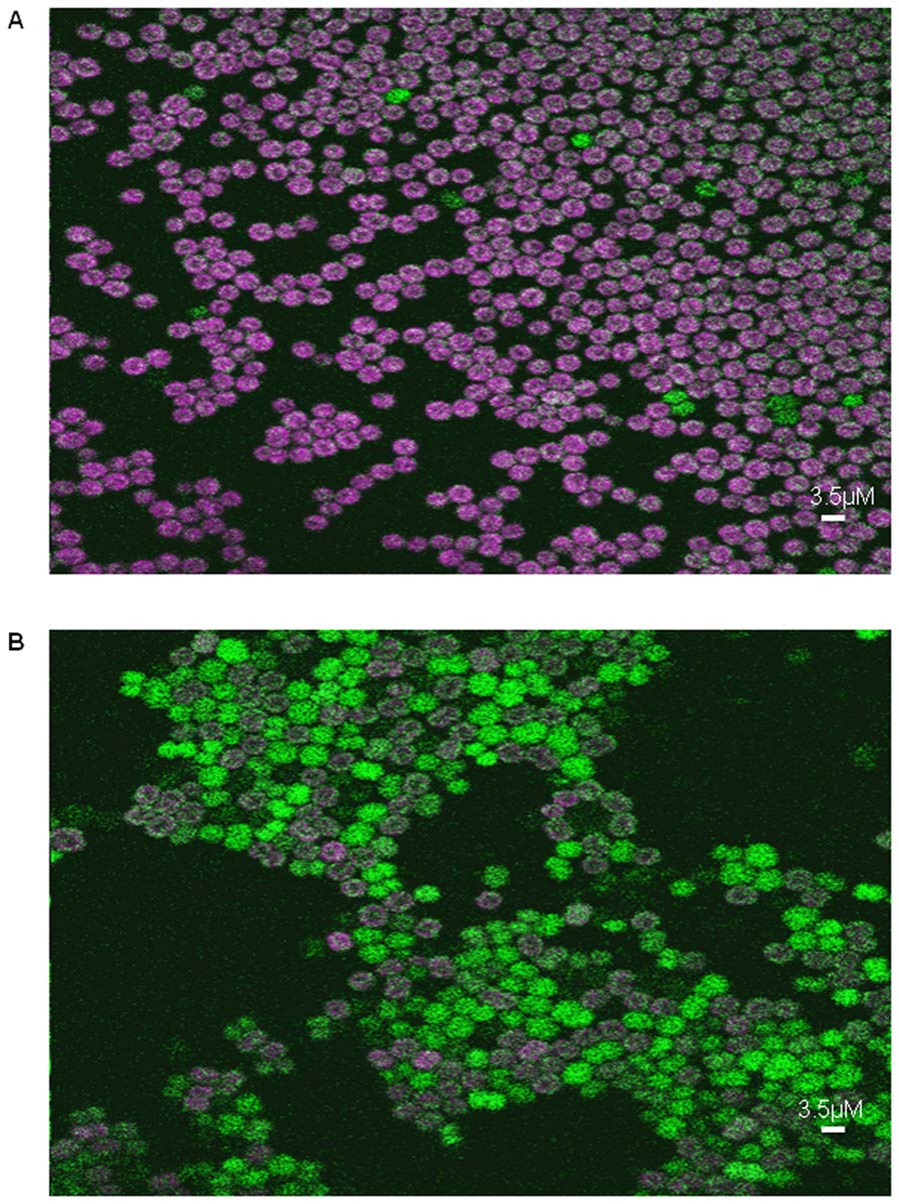
Laser scanning confocal microscopy images of wild type and Δ*slr1768* cells grown under high light. Cells were grown under high-light (130 µmol m^−2^s^−1^) for 48 hrs. Chlorophyll *a* fluorescence is shown in purple and auto fluorescence from predominantly dead and dying cells is green. Chlorophyll *a* fluorescence was collected over the emission wavelength range 670–720 nm. Green auto fluorescence was collected at 500–527 nm. A significant loss in fluorescence was observed in the Δ*slr1768* cells (B) compared to the wild-type (A).

Whole cell absorption spectra for Δ*slr1768* and wild-type cells showed a slight reduction in mean pigment per cell (lower Chl *a* absorbance/per cell scattering at 750 nm) in the mutant compared to the wild-type under normal growth conditions (30 µmol m^−2^s^−1^) (Figure 1 A, C, E). A reduction was seen in the absorption of Δ*slr1768* cells compared to the wild type at wavelengths of 625 and 686 nm corresponding to phycocyanin and chlorophyll respectively. This reduction in pigment was related to light intensity, with the Δ*slr1768* cells having a much greater reduction in pigment per cell when grown under elevated light conditions (130 µmol m^−2^s^−1^) (Figure 1 B, D, F). The growth rate of Δ*slr1768* was also affected conditionally; under normal light conditions Δ*slr1768* had a slightly reduced growth rate compared to the wild-type (Figure 2). However, under elevated light conditions Δ*slr1768* demonstrated a marked reduction in its growth rate (Figure 2). In fact, after the second doubling, at approximately 40 hours of cultivation, growth stopped altogether. Cell counts from 3 independent confocal microscopy experiments confirmed that a large proportion of Δ*slr1768* cells had lost fluorescence (Figure 3B) from the photosynthetic pigments at this point (91%), however a small proportion of cells still looked normal (9%) (Figure 3B), compared to the wild-type cells (Figure 3A). The pigment-free cells did not divide. Under low light conditions a larger proportion of cells looked normal (68%) compared to cells which had lost auto-fluorescence from the photosynthetic pigments (32%) (data not shown).

### Photosynthetic capability is maintained in *Δslr1768*

**Figure 4:**
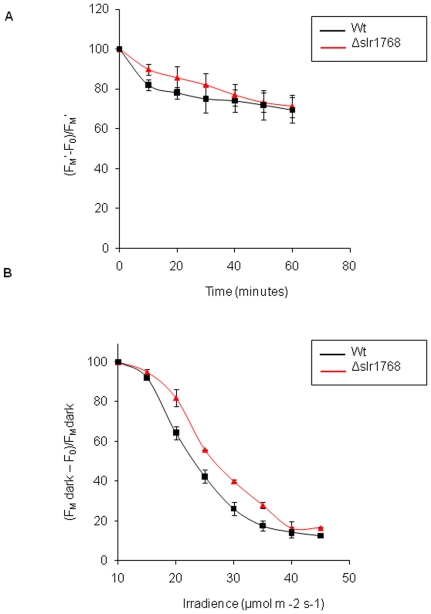
Photosynthetic performance of Δ*slr1768* cells in comparison to wild-type *Synechocystis*. Graph A, maximum photochemical efficiency in the dark after continuous illumination of 1000 µmol m^−2^ s^−1^, quantifying levels of photoinhibition. Graph B, actual quantum yield of photosystem II photochemistry in the light under increasing actinic irradiances. Cells were grown in high light (see Materials and Methods for details).

**Figure 5:**
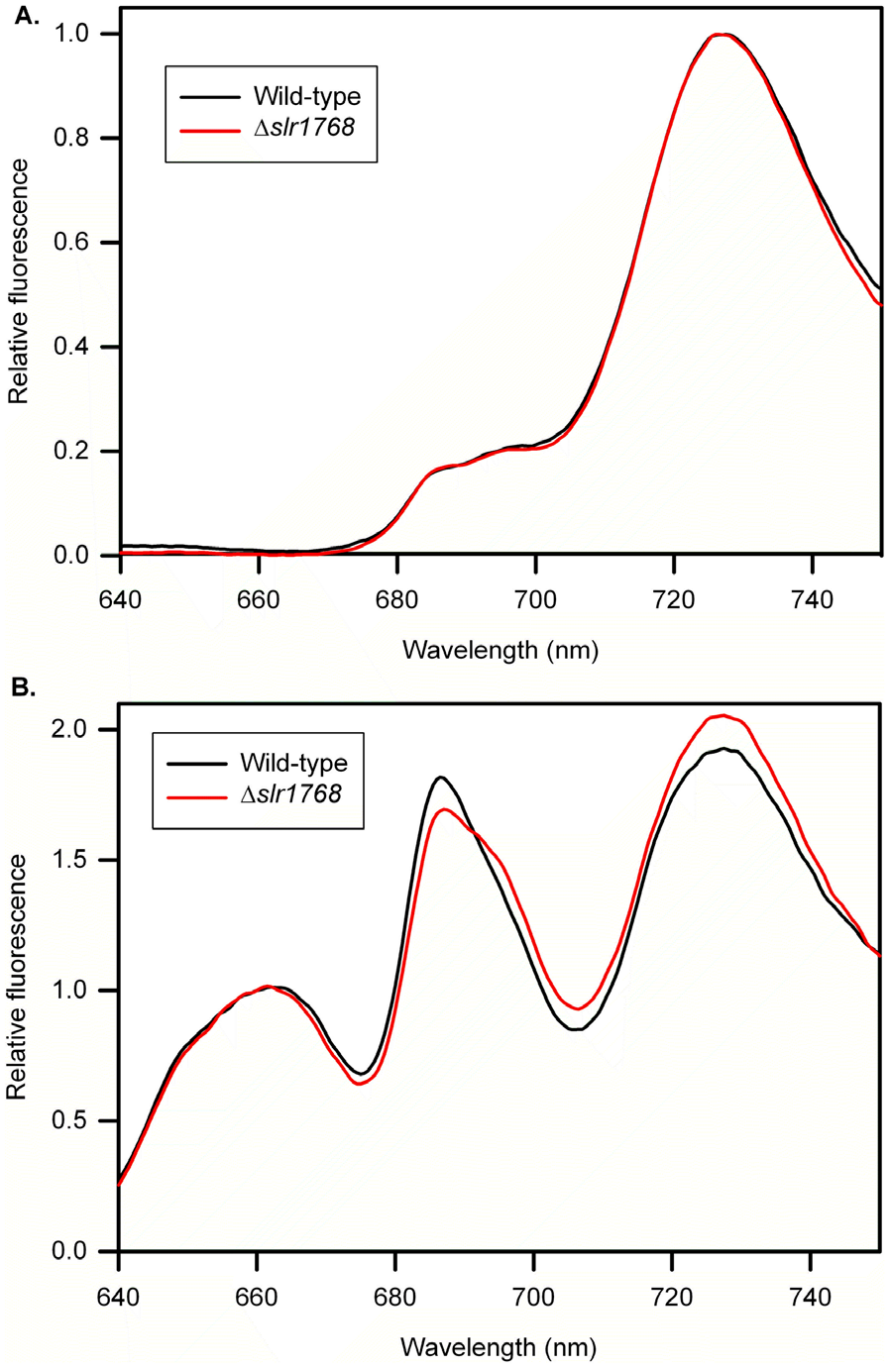
Fluorescence emission spectra measured at 77K. Spectra recorded for frozen suspensions of cells grown in high light (see Materials and Methods for details). Spectra are means from 3 separate samples, normalised at 725 nm (in A) or 665 nm (in B). A: excitation at 435 nm, mainly exciting chlorophyll *a*. B: excitation at 600 nm, mainly exciting phycocyanin.

Room temperature chlorophyll *a* fluorescence was used to measure the maximum photochemical efficiency of photosystem II (PSII) in the dark, (F_m(dark)_ – F_0_)/F_m(dark)_, and the quantum yield of PSII during continuous actinic irradiance, (F_m'_ – F_0_)/F_m'_. The measurements were normalised to the quantity of chlorophyll so as not to bias against the obvious loss of thylakoid membrane in Δ*slr1768*. Figure 4A shows the measurements of (F_m(dark)_ – F_0_)/F_m(dark)_ for wild-type and mutant cells which were initially grown under high light 130 µmol m^−2^⋅s^−1^ and then exposed to moderately high saturating irradiance (1000 µmol m^−2^ s^−1^). The fact that both the wild-type and Δ*slr1768* respond similarly to saturating light treatment indicates that the mutant is able to resist photoinhibition. The quantum yield of PSII reflects the efficiency of electron transport of PSII under constant illumination. Figure 4B indicates that Δ*slr1768* is capable of comparable levels of photosynthetic electron transport when compared to the wild-type strain over a range of light intensities. In addition, we recorded fluorescence emission spectra at 77K for cultures of wild-type and Δ*slr1768* cells grown under high and low light. The spectra show characteristic peaks for PSII at 685 and 695 nm, for PSI at 725 nm and for allophycocyanin at 665 nm (not seen with chlorophyll excitation at 435 nm). The spectra for high-light grown cells (Figure 5) are almost identical for wild-type and Δ*slr1768* cells, showing similar PSII/PSI fluorescence ratios and peak positions. This confirms that the photosynthetic apparatus is assembled normally in those Δ*slr1768* cells that retain pigment. Spectra for low-light grown cells (not shown) show slightly higher PSII/PSI fluorescence ratios in *Δslr1768*, but otherwise show no significant differences.

### Disruption of the *slr1768* gene leads to major changes in thylakoid membrane formation

**Figure 6:**
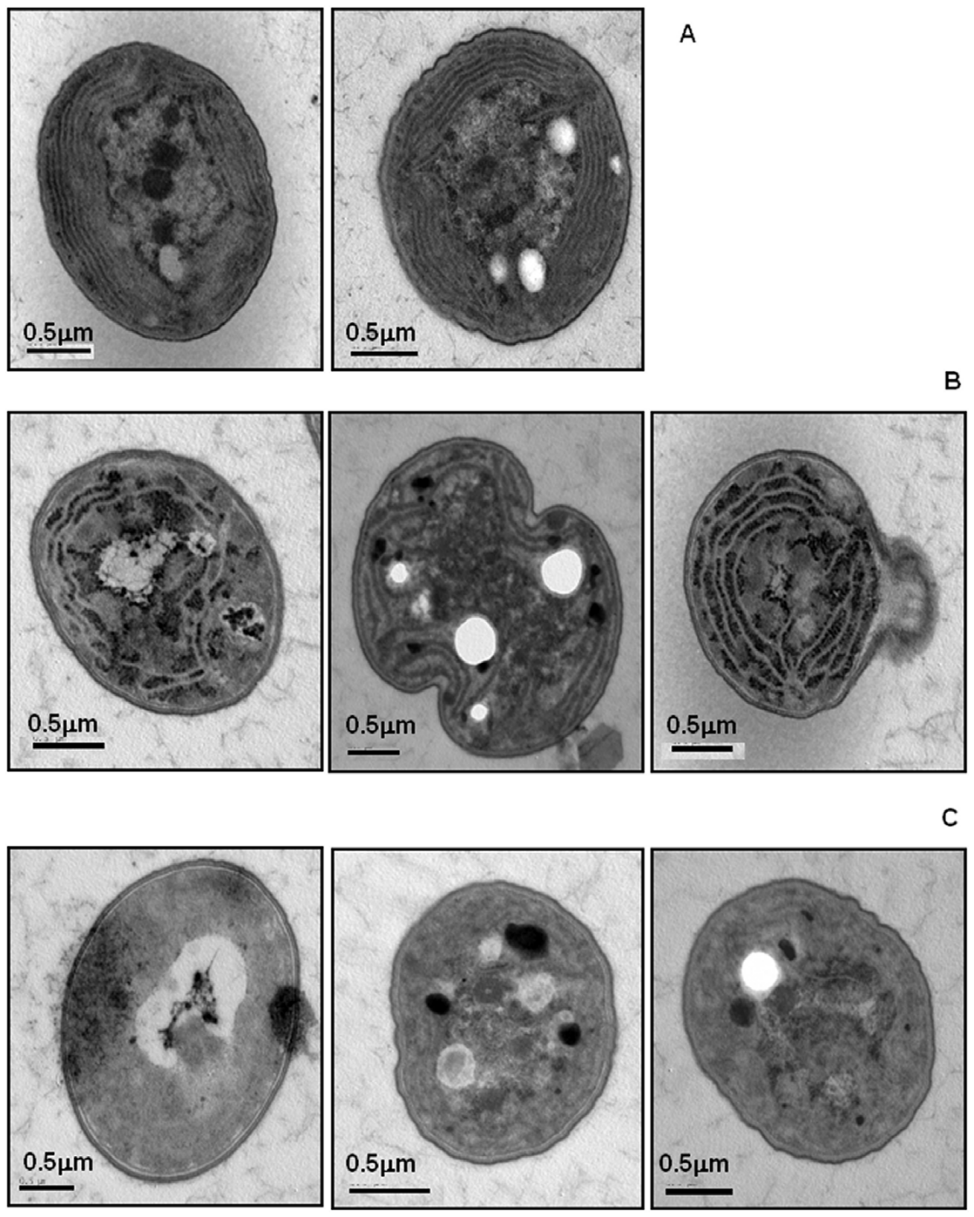
Disruption of *slr1768* leads to a loss of thylakoid organisation and thylakoids. A. Electron micrographs of two typical wild-type cells. B. Four Δ*slr1768* cells that demonstrate a breakdown in thylakoid organisation, C. Examples of Δ*slr1768* cells which lack thylakoid membranes altogether. Both cultures were grown in BG11 media under high light (130 µmol m^−1^ s^−1^) for 60 hrs.

As photosynthetic performance in those cells which retained their pigment was unaffected by the mutation we next determined if the thylakoids were intact and if the loss of pigment content could be attributed to a loss of the thylakoids. Ultrathin sections were prepared from both wild-type and Δ*slr1768* cells grown under both low light (30 µmol m^−2^s^−1^) and high light (130 µmol m^−2^ s^−1^). Figure 6 shows the TEM micrographs for wild-type and Δ*slr1768* cells grown under high light. In wild-type *Synechocystis* cells the thylakoids are well organised in concentric structures that follow the contour of the cell surface (Figure 6A). However, in the mutant two distinct groups of cells can be seen: in a small minority of cells (about 3%) the cells have distinct thylakoids similar to the wild-type, while the remainder and vast majority of the cells are as shown in panels B and C (Figure 6). These cells have either reduced thylakoids, which lack the uniform structure seen in the wild-type cells or they completely lack any internal membrane structure. This was further confirmed by confocal fluorescence imaging, which demonstrated a mixture of normal cells interspersed with non-growing cells, 24 hr growth experiments demonstrated that the cells failed to divide and completely lacked photosynthetic pigments (data not shown). The cells grown in low light conditions also had either reduced thylakoids or cells which lacked thylakoids altogether (data not shown); however these were not as numerous as in the cultures grown under high light.

### Thylakoid loss in Δ*slr1768* is not a generalised stress response

**Figure 7:**
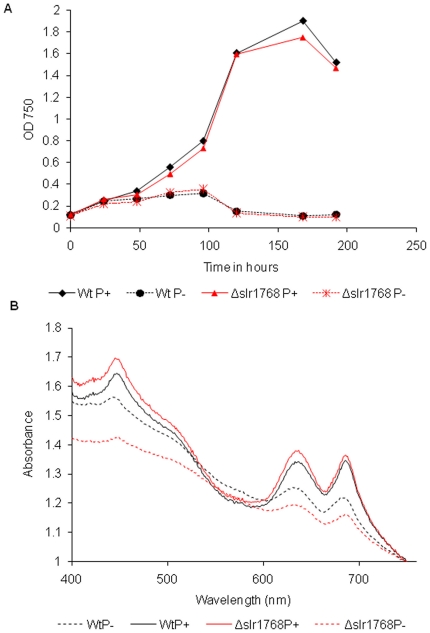
Photoautotrophically grown wild-type *Synechocystis* and Δ*slr1768* cultures under phosphate deprivation. (A) Graph shows growth of wild-type and Δ*slr1768* cells grown in BG11-P and BG11 over 192 hours under low light (30 µmol m^−2^s^−1^). (B) Whole-cell absorption spectra of wild-type and Δ*slr1768* cells grown under low light (30 µmol m^−2^s^−1^) in BG11-P and BG11 for 90 hours.

To test the specificity of the Δ*slr1768* phenotype, we compared the effects of high-light treatment in Δ*slr1768* with the effects of phosphate deprivation, a stress treatment which eventually leads to loss of pigment and thylakoid membranes in wild-type cells. Parallel cultures of wild type and Δ*slr1768* cells were matched for cell density (A_750_), gently pelleted and washed three times in BG11 that contained no source of phosphate (BG11-P). The cells were then re-suspended in BG11-P and grown at 30°C under 30 µmol m^−2^s^−1^ illumination. At this light intensity, the growth rate under phosphate deprivation of Δ*slr1768* was comparable to that of the wild-type (Figure 7A), and pigment loss was not accelerated in Δ*slr1768* as compared to the wild-type (Figure 7B). This indicates that the Δ*slr1768* mutation does not impose a "generic" background stress which leads to greater susceptibility to any stress treatment. Rather, the Δ*slr1768* mutation leads to quite specific problems with maintaining the thylakoid membranes and the photosynthetic apparatus under high-light conditions.

### Phylogenetic Analysis of the *slr1768* gene

An interrogation of the genome sequence of the cyanobacterium *Synechocystis sp.* PCC 6803 using the HflK and HflC sequences from *E. coli* revealed the presence of four HflK/C homologues Slr1106, Slr1768, Slr1128 and Sll0815. To search for further orthologs across the prokaryotic and eukaryotic kingdoms, the Slr1768 sequence was used to search the KEGG and Cyano databases. Each species was selected on the following criteria: sequence similarity to the *hflK* and *hflC* gene from *E. coli* (where the role of both HflK and HflC has been comparatively well characterised), and the presence of an HflK/C/band 7 domain. Phylogenetic analysis using MrBayes [28], [29] was performed on the nucleotide sequences, thereby determining key interrelationships. This revealed that the proteins are all related but highly divergent. We tentatively identified four approximate groupings (data not shown). The mammalian sequences clustered very tightly together demonstrating that they are highly conserved. Interestingly, both red algal and cyanobacterial sequences can also be found linked to this group. The HflK/C homologues from *Synechocystis* did not group together, Slr1106, which is defined as a prohibitin, groups with other potential prohibitin homologues, while Slr1128 is more closely related to the HflC and HflK homologues defined by Group C. This group also contains the plant orthologs. Perhaps most surprising is the inclusion of Slr1768 in a group which comprises cyanobacteria, green sulphur bacteria and *Methanococcus aealous*, a member of the Archea. The two closest relatives to *slr1768* are hypothetical genes from *Cyanothece* and *Nostoc*. Both are band seven proteins, and also, interestingly, both contain an HflC domain.

## Discussion

Our study demonstrates an important role for the prohibitin homologue Slr1768 in the maintenance of thylakoid membranes and the photosynthetic apparatus in the cyanobacterium *Synechocystis sp*. PCC 6803. Deletion of *slr1768* results in the complete loss of thylakoid membranes and chlorophyll in a proportion of cells in the culture. Two cyanobacterial genes have previously been identified whose deletion results in substantial loss of the thylakoid membrane system. These are *vipp1* and *alb3*, which, like *slr1768*, encode plasma membrane proteins [3]. However, the mutant phenotypes indicate that the role of Slr1768 is significantly different from both Vipp1 and Alb3. Complete deletion of the *vipp1* (*sll0617*) and *alb3* (*slr1471*) genes has never been achieved, as surviving cells always retain some wild-type loci among their multiple chromosome copies [18] [9]. This is consistent with the idea that both gene products are essential for the biogenesis of the thylakoids and the photosynthetic apparatus. However it remains uncertain whether loss of Vipp1 and Alb3 in cyanobacteria primarily effects the production of the thylakoid membranes themselves. It is possible that either protein could be essential for production of photosynthetic complexes, with loss of the thylakoid membrane system being a secondary consequence of loss of the photosynthetic apparatus. Alb3 appears to play an essential role in membrane insertion of the D1 protein of Photosystem II [19], and recent evidence implicates Vipp1 in the biogenesis and stability of Photosystem I in *Synechocystis* sp. PCC 6803 [14].

In contrast to *vipp1* and *alb3* we show that *slr1768* can be completely deleted, with full segregation of the chromosomes. However, although the null mutant is genetically homogeneous, the phenotype is clearly stochastic. While a proportion of cells in the culture show complete loss of thylakoid membranes and chlorophyll, the remaining cells appear completely normal in terms of their pigment and thylakoid membrane content and photosynthetic performance. At low growth light intensity, only a small proportion of cells are affected by the mutation. At higher light intensities, the majority of cells are affected. Unsurprisingly, the cells that lose thylakoid membrane and pigment also cease to grow and divide. Slr1768 is clearly not an essential biogenesis factor for the thylakoid membranes and the photosynthetic apparatus, since some cells of the null mutant appear completely normal in terms of their photosynthetic properties. Rather, there appears to be a failure of some safety mechanism that prevents the occasional self-destruction of the entire thylakoid system. Cells of the null mutant must be continually at risk of loss of the photosynthetic apparatus and death: the probability of this event increasing with light intensity. What triggers the breakdown of the thylakoid membrane system in the mutant? The frequency of the event is clearly increased at moderately high light intensities, but we see no indication of a pre-existing perturbation of the photosynthetic apparatus. Our measurements clearly show that those cells which retain their pigment are normal in their photosynthetic properties, and 77K fluorescence spectra (normally a sensitive indicator of the state of the photosynthetic complexes) show no significant differences from the wild-type. We have not yet been able to fully determine the sequence of events when the thylakoid system breaks down, but electron micrographs do show some cells that appear to be in the process of breakdown: they have a reduced population of thylakoids with perturbed organisation. Since we cannot detect any equivalent partial loss of photosynthetic activity, this suggests that the primary breakdown event is loss of the membrane system, with loss of the photosynthetic apparatus following. The stress leading to increased frequency of breakdown at higher light intensities may be due either to both the increased requirement for repair of the photosynthetic apparatus under these conditions, or the increased rate of thylakoid membrane biogenesis required when the cells are growing rapidly at moderately high light intensities.

Boehm *et al*., (2009) recently reported a *slr1768* knock-out in *Synechocystis* sp. PCC 6803. They generated *slr1768* mutants in the both the glucose tolerant strain and the wild-type strain. The SPFH domain was disrupted by insertion of an antibiotic resistance cassette. PCR analysis confirmed that the mutant was fully segregated in both the glucose tolerant and the WT strain; however they were unable to find a phenotype for this mutant in both strains and concluded that Slr1768 is not essential for cell viability [30]. The differences seen between our Δ*slr1768* strain and the Δ*slr1768* strain created by Boehm *et al*., (2009) could be attributable to a number of factors, including the method of gene deletion. We constructed a full in frame deletion, removing the entire gene, however Boehm *et al*., (2009) made an insertional mutation which left 256 bp at the N terminus. This residual N terminal sequence could result in a partially functional protein being expressed, which requires further investigation.

In bacteria, the SPFH family is represented by the HflK and HflC proteins, both prohibitin homologues. The SPFH domain is thought to have arisen either independently through convergent evolution or through lateral gene transfer in Prokaryotes [31]. In *E. coli* the HflK and HflC proteins form a complex that regulates the actions of a membrane bound zinc metalloprotease, FtsH. Recent research by van Bloois *et al*., (2008) has demonstrated that in *E. coli*, YidC (an Alb3 homologue), is associated with the HflK/C and the FtsH complex [24]. YidC may also function as a chaperone protecting new membrane proteins from degradation by FtsH. It has been suggested that this may also occur within the mitochondria between the YidC and FtsH homologues Oxa1 and Yme1 [32]. The YidC/Alb3/Oxa1 homologues are functionally conserved in the process of membrane protein insertion. Phylogenetic analysis revealed that Slr1768 is related to HflK and HFLC, but it does not group directly with the HflK/C homologues, instead forming a small group with hypothetical genes from *Cyanothece* and *Nostoc*, suggesting that these proteins may have diverged over time adapting to a specific role within the bacteria. Bioinformatic analysis demonstrated that Slr1768 is not widely prevalent in the cyanobacterial genomes currently available on Cyanobase and Kegg, suggesting that it may not be a typical prohibitin represented in cyanobacteria. It is interesting to note that Slr1768 still has an HflC domain, thus making it part of the SPFH super-family. SPFH domains all tend to share common features in that they have a tendency to oligomerise forming membrane complexes and potentially associating with lipid rafts [33]. It is therefore very plausible that Slr1768 could have a regulatory role in thylakoid biogenesis and maintenance under light stress, similar to the roles of other SPFH proteins.

## Materials and Methods

### Bacterial strains and media

*Synechocystis* sp. PCC 6803 (WT) (not the glucose tolerant strain) was grown photoautrophically in BG-11 medium [34] at 30°C under 50 µmol m^−2^ s^−1^ white light in glass flasks, with continuous shaking. For low light conditions cells were grown at 30°C under 30 µmol m^−2^ s^−1^ white light and for high light cells were grown at 30°C under 130 µmol m^−2^ s^−1^ white light. At the maximum cell concentrations used, light intensity in the middle of the flask was decreased by a factor of about 2 due to self-shading. For phosphate deprivation cells were grown in BG-II media were the K_2_HPO_4_ (0.18 mM) had been replaced with an equimolar solution of KCL (0.18 mM), giving a final Cl^-^ concentration of 0.31 mM.

### Transformation of *Synechocystis* sp. PCC 6803

*Synechocystis* sp. PCC 6803 cells were transformed according to [35]. Briefly a culture in exponential growth was harvested and washed with fresh BG-11 and re-suspended to give a final concentration of 1×10^9^ cells per ml^−1^. Approximately 10 µl of plasmid DNA was then added to 150 µl of cells and incubated at 50 µmol m^−2^⋅s^−1^ white light at 30°C for 1–5 hours before being spread onto BG-11 plates. The plates were then incubated under 50 µmol m^−2^ s^−1^ white light at 30°C until confluent green growth was observed (approximately 16 hours). Increasing amounts of spectinomycin were then added, cells were further grown on selective plates containing a final concentration of 50 µg ml^−1^ spectinomycin.

### Sequence analysis

The nucleotide and protein sequences for the hypothetical gene *slr1768* were used to identify orthologous sequences in both the NCBI database (http://www.ncbi.nlm.nih.gov), and the KEGG database (http://www.genome.jp/kegg/). Only sequences with significant E-values were chosen for analysis. HflC motifs and band 7 motifs were identified using CDART (NCBI), MotifScan and SMART (http://www.expasy.org/).

### Phylogenetic analysis

Nucleotide sequences from orthologs of the *slr1768* gene were aligned using ClustalW (http://www.ebi.ac.uk/clustalw), specifically, amino acid sequences were aligned with ClustalW and then the corresponding DNA alignment was determined by reverse translation using the original DNA sequences. Phylogeny was inferred using the Markov chain Monte Carlo package Mr Bayes (http://mrbayes.csit.fsu.edu/) with the HKY model that is parameterised by nucleotide frequencies, branch lengths and the transition-transversion ratio κ. Convergence was confirmed using a multiple run methodology [36].

### Generation of a *slr1768* disruption mutant in *Synechocystis sp*. PCC 6803

The *slr1768* mutant was generated as detailed in the REDIRECT manual [37], with minor modifications. The protocol, plasmids and strains were provided by PBL Biomedical Laboratories. The forward *slr1768*F (ACGACTCAAGTCCACATAGG) and the reverse *slr1768* (CACCAGGGTGGAAGCTAAAC) primers were used to amplify a 3 kb region, which included the *slr1768* gene flanked by 1 kb either side, to assist with homologous recombination. The 3 kb PCR product was cloned into the pGEM T-easy vector (Promega) as detailed in the Promega manual. Successful transformants were screened via PCR and sequenced using the T7 and S6 primer (Promega).

### Chlorophyll *a* concentration determination

1 ml of *Synechocystis* culture was centrifuged for 1 minute at 9500 g, the supernatant was removed and 1 ml of methanol was added. The pellet was then re-suspended thoroughly by vortexing, and left at 60°C for 5 minutes to extract the pigment. The cell debris was then pelleted at 9500 g for 1 minute and the supernatant removed. Concentrated samples were diluted in methanol. Chlorophyll *a* concentration was measured in a spectrophotometer in cuvettes using methanol as a blank at OD_665_. Chlorophyll *a* concentrations were determined by; OD_665_ × dilution factor ×13.42 = µg ml^−1^ chlorophyll *a* [38].

### Whole cell absorbance spectrum

Whole cells were scanned for absorption in a UV 500 spectrophotometer. Absorption was measured between 400 nm and 760 nm and cell density was normalized at 750 nm using the Vision 32 software.

### Whole cell 77 K fluorescence emission spectra

77K fluorescence emission spectra were measured in a Perkin-Elmer LS50 luminescence spectrometer. Cells were adjusted to a chlorophyll *a* concentration of 5 µg ml^−1^ injected into 4 mm diameter silica tubes, dark adapted and frozen in liquid nitrogen. The excitation and emission slit widths were 5 nm. Measurements were taken with 600 nm excitation (phycocyanin absorption band) and 435 nm excitation (Soret absorption band for Chl *a*).

### Chlorophyll *a* fluorescence measurement

Chlorophyll fluorescence was measured at room temperature using a pulse amplitude-modulated fluorometer (Hansatech, King's Lynn UK), normalised to 5 µg chlorophyll ml^−1^. Maximum photochemical efficiency was calculated as (F_M'dark_ – F_0_)/ F_M'dark_. Cells were measured on a chlorophyll basis of 5 µg chlorophyll ml^−1^. Samples were dark adapted for 5 minutes prior to measurement, then given a saturating pulse of light to give maximal fluorescence in the dark, F_M'dark_. The photochemical efficiency was measured under high actinic light (1000 µmol m^−2^ s^−1^), the F_M'dark_ parameter providing a measurement of PSII efficiency under photoinhibitory light. Saturating pulses measuring F_M'dark_ were taken after dark adaptation at 0, 10, 20, 30, 40, 50 and 60 minutes of actinic light (1000 µmol m^−2^ s^−1^). The photochemical efficiency was calculated as (F'_m_- F_S_)_/_F'_m_. Cells were measured on a chlorophyll basis of 5 µg chlorophyll ml^−1^ and dark adapted for 5 minutes. F_m_ was measured after 20 minute periods, at actinic irradiances of 17, 41, 85, 150, 240, 365, 520 and 720 µmol m^−2^ s^−1^.

### Transmission Electron Microscopy

Wild-type *Synechocystis* sp. PCC 6803 and Δ*slr1768* strains were collected and fixed in an equal volume of 6 % glutaraldehyde, 0.2 M sodium cacodylate buffer, pH 7.2, for 2 hours. After fixation, the cells were washed in distilled water three times for 10 minutes. The fixed cells were then incubated in 1 % osmium tetroxide, 0.1 M sodium cacodylate buffer, pH 7.2, containing 0.8 % potassium ferricyanide for 60 minutes followed by washing in distilled water three times for 10 minutes. The cells were then embedded in 2 % agar and incubated in 70 % ethanol for 30 minutes at room temperature followed by a further overnight incubation at 4°C. After continued dehydration, the cells were embedded in Spurr's resin [39], and 60–90-nm sections were cut using a Reichert ultracut E ultramicrotome. Ultrathin sections were then counterstained with 2 % aqueous uranyl acetate followed by Reynolds lead citrate and viewed using a Jeol 1220EX transmission electron microscope. Images were recorded using a Gatan Dual Vision™ 300W digital camera [18].

### Confocal Microscopy

Cells were immobilised by absorption onto blocks of BG-II agar in a custom built sample holder using a PCM2000 laser-scanning confocal microscope (Nikon). Cholorophyll fluorescence was excited with the 488 nm band of a 100 mW argon laser, and selected using a Schott RG665 filter transmitting wavelengths above about 665 nm. A 20 µM confocol pinhole was used with a 60x (NA1.4) objective lens, giving resolution in the Z-direction of about 1.3 µM.
